# From Theory to Practice: Artificial Intelligence (AI) Literacy Course for First-Year Medical Students

**DOI:** 10.7759/cureus.70706

**Published:** 2024-10-02

**Authors:** Hunter Levingston, Max C Anderson, Monzurul A Roni

**Affiliations:** 1 Health Sciences Education and Pathology, University of Illinois College of Medicine, Peoria, USA; 2 Curriculum Operations, University of Miami Miller School of Medicine, Miami, USA

**Keywords:** artificial intelligence, chatbot, course, curriculum, medical education

## Abstract

Artificial intelligence (AI) is rapidly transforming healthcare by enhancing diagnostics, personalized medicine, and clinical decision-making. In medical education, AI chatbots have the potential to be used as virtual tutors or learning assistants. Despite AI's growing impact, its integration into medical education remains limited. AI is not a standard component of medical curricula, which could leave many future healthcare professionals unprepared for an AI-driven workplace. To address this significant gap, this editorial describes the development of a mini-course to integrate AI training for first-year medical students. The course was focused on the fundamentals of AI, prompt engineering, practical applications of chatbots as learning assistants, and ethical use of generative AI.

## Editorial

Artificial intelligence (AI) is rapidly transforming healthcare and educational landscapes. In healthcare, it is used to enhance radiology, obtain faster and more accurate diagnoses, screen cancers, facilitate clinical trials or robotic surgery, and make predictions from electronic health records (EHRs). Integration of AI into the healthcare system has led to improved patient care, reduced medical errors, and optimized treatment plans. Due to its wide applications in clinical practice, there is a growing concern in the medical community that clinicians who use AI will supplant clinicians who do not use it. Like healthcare, AI has transformed medical education. AI-based tools such as chatbots can provide personalized learning experiences by adapting to the knowledge level of each student. By using AI as a virtual tutor or learning assistant, medical students can get instant personalized feedback and additional resources and assess their readiness for examinations by creating quizzes. AI can help in developing clinical reasoning skills by presenting students with clinical case simulations. It can be utilized to provide conversational pedagogy to help students form answers to open-ended questions and practice communication skills. AI-based tools have shown remarkable success in passing USMLE Step exams, making them valuable teaching and learning tools. Despite the significant impacts of AI, the inclusion of this new technology in medical education remains underexplored.

Evidence suggests that about half of medical students are using generative AI chatbots like ChatGPT for summarizing, revising, researching, or learning new material [1]. Due to its ability to analyze and summarize a large amount of content, AI can potentially offer a more efficient way of learning. Medical education, like other health professions education, continues to expand in curricular and co-curricular activities. Student doctors juggle competing demands for their time to complete pre-course work, exam preparation, and other academic activities. With proper training, AI can be utilized to cut down the time needed to prepare for classes and exams. However, many medical trainees do not receive sufficient training on AI technology in their medical curriculum [2]. Without training on the responsible use of AI, the users are at risk of violating data privacy and copyright and relying on confabulated data produced by generative AI. To address this significant gap in AI training, this editorial describes the development of a mini-course tailored for medical students and how it can be provided as an online resource that can be adapted by other institutions.

**Figure 1:**
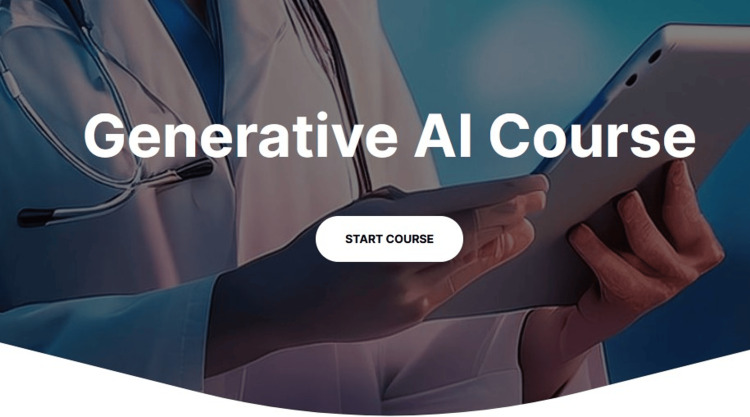
Generative AI course for medical students. The mini-course is publicly available through a link in the references [3].

We developed this course (Figure 1) for first-year medical students at the University of Illinois College of Medicine, Peoria (UICOMP) [3]. This mini-course was offered as optional asynchronous training. The self-paced course is accessible by anyone with an internet-connected device. As AI technology continues to evolve rapidly, training programs developed today may become outdated tomorrow. To overcome this limitation, AI courses require continuous updates and revisions. We used an eLearning authoring tool (Articulate Rise 360) to create this course which allowed us to make necessary updates ensuring the content remains relevant and useful for students.

There is little consensus about which AI topics should be taught at medical schools. Teaching AI-related computer engineering and data science is beyond the scope of the medical curriculum, specifically at the preclinical stage. Four areas were recommended for the AI curriculum of medical students including foundational AI, practical AI, experimental AI, and ethical AI [4]. It was suggested that the AI training be spread out in the preclinical and clinical stages based on relevancy. For example, preclinical years should focus on AI terminology, applications in medicine, data privacy, and features of AI tools. Clinical years should focus on the role of AI in diagnostic imaging, health data management, and patient communication [4]. Further guidance for developing an AI curriculum for clinical care was provided by Russell et al. [5] who identified six clinical competencies which included basic knowledge, social and ethical implications, AI-enhanced clinical encounters, evaluation of AI tools, workflow analysis of AI tools, and practice-based learning.

Based on the domains identified by previous studies [4,5], our AI course covered the fundamentals of AI, prompt engineering, practical applications of chatbots as learning assistants, and ethical aspects of generative AI. Much of the content was presented with a series of short videos (1-8 minutes). To prepare the first-year medical students on how to leverage generative AI for study, the prompt engineering section focused on formulating better prompts to create study aids such as summary tables, mnemonics, flowcharts, clinical simulations, board-style multiple-choice questions, etc. The prompt examples were also provided as a separate handout. In addition to chatbots, other AI tools that are useful for scientific literature review, flashcard creation, and PDF analyses were covered in the course. The ethical and legal issues related to generative AI use were presented at the end of the course which included academic integrity, equity related to access to advanced language models, loss of jobs, environmental impact, data privacy, copyright issues, and accountability. Although there are many AI chatbots available, the course primarily focused on the use of ChatGPT (OpenAI, USA) and Copilot (Microsoft, USA). Both chatbots are built on the same GPT architecture and they have the largest number of users. The estimated time to complete the course was 90 minutes. Overall, preliminary feedback from the course participants was highly positive.

It is noteworthy to mention here the arguments against providing AI training to students in the pedagogy circle. Firstly, the current medical curriculum is overcrowded with basic, clinical, and social sciences. There is little time for familiarizing medical trainees with a new AI curriculum. As board examinations do not test new technologies, AI is often integrated into the curriculum in a superficial sense. There is no formal framework to incorporate AI in the medical school curriculum. The lack of structured training poses a challenge for the student doctors who will be engaged in AI-driven tools in clinical practice. Proposals to include AI as an additional curricular element or course struggle to gain traction in an already dense medical curriculum. Rather than ignoring AI, a balanced approach could be to at least offer workshops or online training modules like our mini-course as supplementary resources for medical students.

A secondary argument against providing AI training to students is that it can open the door to cheating and plagiarism in assignments. An additional concern is the development of AI dependency in students who may use AI to find solutions for every problem without developing problem-solving skills. These arguments can be countered by fostering a culture of responsible and ethical AI use. Responsible AI use among students can be fostered with the adoption of AI training at the early stages of education and appropriate AI usage policies. We are in favor of cautious optimism and critical appraisal of AI applications in medical education. While most of the AI training in medical education has predominantly focused on educators, we believe training medical students and health professional trainees is equally vital.

In summary, future clinicians must be skilled in data input and interpreting AI-derived treatment policy in AI-dominated workplaces. Medical schools should focus on integrating AI into medical education, with an emphasis on the technical, ethical, and legal aspects of its use. By developing robust and adaptable training programs, we can ensure that future healthcare professionals are well-equipped to leverage these innovative tools in their education and clinical practice. We anticipate this mini-course will provide sufficient know-how for both students and faculty.
